# What do positive and negative experiences of patients, relatives, general practitioners, medical assistants, and nurses tell us about barriers and supporting factors in outpatient palliative care? A critical incident interview study

**DOI:** 10.3205/000284

**Published:** 2020-09-18

**Authors:** Stephanie Stiel, Helen Ewertowski, Olaf Krause, Nils Schneider

**Affiliations:** 1 Institute for General Practice, Hannover Medical School, Hannover, Germany

**Keywords:** health care research, palliative care, Critical Incident Technique, primary palliative care

## Abstract

**Background:** The strengthening of the general practitioners’ (GPs’) role in palliative care (PC) has been identified as a top priority in order to improve PC in Germany. This study aims at exploring positive and negative experiences in PC in Germany from the perspectives of patients, relatives, and health care professionals in a primary care setting.

**Methods:** Between March 2017 and August 2017, a total of 16 interviews with patients, relatives, GPs, medical assistants, and nurses were conducted. The Critical Incident Technique (CIT) was used to explore factors that influence excellent versus undesirable events in PC provision. Two researchers independently defined and counted critical incidents (CIs) from interview transcripts, performed a thematic analysis, and clustered the CIs into dimensions.

**Results:** In summary, 16 interviews contained 280 CIs, divided into 130 positive and 150 negative CIs. The thematic analysis resulted in seven content domains, with each including positive and negative CIs, respectively: 1) way of care provision, 2) availability of care providers, structures, medication, and aids, 3) general formal conditions of care provision, 4) bureaucracy, 5) working practices in health care teams, 6) quality and outcome of care provision, and 7) communication.

**Conclusions:** The results raise awareness for the aspects that lead to successful or undesirable PC experiences, observed from different perspectives. They open up the potential for primary PC improvement. Future research will facilitate development and implementation of more tailored interventions in order to improve generalists’ PC.

## Zusammenfassung

**Einleitung:** Um die Palliativversorgung in Deutschland zu verbessern, wurde die Stärkung der Rolle von Allgemeinmedizinern in der Palliativversorgung als höchste Priorität eingestuft. Das Ziel dieser Studie ist das Erforschen positiver und negativer Erfahrungen mit Palliativversorgung in Deutschland aus der Perspektive von Patienten, Angehörigen und medizinischen Fachkräften in der Grundversorgung.

**Methoden:** Von März 2017 bis August 2017 wurden insgesamt 16 Interviews mit Patienten, Angehörigen, Allgemeinmedizinern, medizinischen Fachangestellten und Pflegekräften geführt. Dabei wurde die Critical Incident Technik benutzt, um Faktoren zu finden, die positive oder unerwünschte Ereignisse in der Palliativversorgung beeinflussen. Zwei Forscher haben unabhängig voneinander Critical Incidents (CIs) in Interviewprotokollen definiert und gezählt, eine thematische Analyse durchgeführt und die CIs in Dimensionen gruppiert.

**Ergebnisse:** Zusammengefasst enthielten 16 Interviews 130 positive und 150 negative CIs. Das Ergebnis der thematischen Analyse waren sieben Inhaltsdomänen, die jeweils positive und negative CIs beinhalten: 1) die spezifische Art der Versorgung, 2) Verfügbarkeit von Diensten, Strukturen, Medikamenten und Hilfsmitteln, 3) formale Rahmenbedingungen der Gesundheitsversorgung, 4) Bürokratie, 5) Arbeitsweisen in Teams der Gesundheitsversorgung, 6) Qualität der Arbeit und Ergebnisse der Versorgung, und 7) Kommunikation.

**Schlussfolgerung:** Die Ergebnisse sensibilisieren für die Aspekte, die zu erfolgreichen oder unerwünschten Erfahrungen mit Palliativversorgung führen, beobachtet aus verschiedenen Perspektiven. Sie erschließen das Potenzial für eine Verbesserung der palliativen Versorgung. Zukünftige Forschung wird die Entwicklung und Implementierung spezifischer Interventionen vorantreiben, damit die von Generalisten ausgeübte Palliativversorgung verbessert werden kann.

## Background

There is a large national and international consensus about generalist palliative care (PC) being delivered by general practitioners (GPs). They play a vital role in caring and supporting severely ill and dying patients. This is irrespective of oncologic and non-oncologic diseases – especially focusing on continuity of care [1], [2], [3]. GPs provide services in different settings such as private homes, nursing homes and hospices [4], [5]. By the current state of scientific knowledge, about 20% of all patients with oncologic diseases and 5% of patients with non-oncologic diseases present a need for specialized PC. In conclusion, the majority of terminally ill patients, even with end-stage diseases, are finding suitable and sufficient support in generalist PC by GPs [6].

Scientific and political initiatives have focused on generalist PC [7], [8]. The empowerment of GPs in PC, as well as ensuring a tight cooperation with PC specialists, have been identified to be a top priority for the further development of PC in Germany. The high relevance of PC for GPs has been embedded in German law since 2015 (statute for reinforcement of hospice and palliative care) [9]. Despite this fact, up to now a transparent, concise definition of the term “generalist PC” and a comprehensive description of the factors influencing GPs’ provision of care are missing. For the following, the authors define generalist PC by GPs as PC approach to ensure a smooth transition between curative and palliative treatment of critically ill and dying patients in order to reduce suffering, to relieve pain and to ensure quality of life. The physical, psychological, social and spiritual needs of patients and their relatives are to be met, and personal, cultural and – where applicable – religious values and convictions are to be treated with sensitivity. Generalist PC is a basic care approach which can be supplied by GPs without specific training in PC, in contrast to specialized PC.

Internationally, in the context of generalist PC research and its practicability, the challenges for GPs in their daily work have been carefully worded. In a systematic review, Mitchell et al. found that patients appreciate if the GP is accessible, takes time to listen, allows patients and carers to express their feelings, and manages symptom relief. On the other hand, GPs express doubt about their competence to perform PC adequately [10]. Based on the work of other peers [8], [9], four main factors that influence the willingness to deliver PC and the quality of GPs’ PC provision have been identified. In conclusion:

1. The proposition of generalist PC varies and is severely dependent on the allocation of care, i.e. there might only be a few GPs in wide-spread rural areas with consequential lack of patients’ provision.
2. An existing lack of knowledge and skills in PC regarding GPs is to be considered [11]. Taking part in further PC training represents an additional hurdle and time conflict.
3. There are also challenges in providing continued care for PC patients. For instance, GPs are only marginally involved in patient care if their patients are simultaneously treated by PC specialists.
4. Additionally, a certain “diffusion of responsibility” can occur between GPs and PC specialists, creating conflicting views of doctors’ roles and responsibilities for treating severely ill and dying patients [12], [13].

Consequently, it is not known how GPs face these challenges of PC provision in their daily routine, and which fostering and inhibiting factors can be found for generalist PC. To analyze fostering and inhibiting factors in more detail, different perspectives on the lived reality of providing and receiving generalist PC can be of great value.

### Study context and aim 

The Federal Ministry of Education and Research in Germany (BMBF) promotes within a plan of action a 5-year project of a junior research group for generalist PC in the setting of a GP surgery (ALLPRAX, Grant No. 01GY1610). The project has started in November 2016. The overriding aim of this project is to improve the circumstances for the implementation of PC provision via GPs. Minor targets are:

1. the exploration and systemic analysis of inhibiting and fostering factors for GPs’ PC provision in four sub-projects (three qualitative, one quantitative) that will lead to one data synthesis,
2. the development of a tailored package of interventions as well as a strategic plan to integrate new processes in daily practice, and
3. the implementation of the newly developed processes and evaluation of their practical use in PC and their impact on patient care. The study protocol is published elsewhere [14].

The article at hand provides results from target 1. The principle research items read as follows: What do patients’, relatives’, GPs’ and medical professionals’ positive and negative experiences tell us about barriers and facilitators to primary PC provision in daily practice? What impact do these barriers and facilitators have on all individuals concerned, and what are the implications on the outcomes of PC delivery?

## Methods

### Study design and study population

In the period between March and August 2017, all n=4 patients, n=4 relatives, n=4 GPs, n=2 medical assistants, and n=2 nurses (summarized here as n=4 medical professionals) were invited to a guided interview following the Critical Incident Technique (CIT). The selection of participants followed the predefined targeted sample size and target groups described in the grant application. The method of CIT was chosen to collect practice-oriented positive and negative lived experiences of generalist PC. In further steps, the CIT results will be summarized with results of two more qualitative sub-projects from study target 1 in a Grounded Theory analysis to gain a profound understanding of barriers and facilitators in PC [14].

Within the predefined number of 16 CIT interviews, the authors considered heterogeneous sampling criteria of all four target groups such as gender, age, rural and urban location, type of general practice (single practice or joint practice), health care experiences of GPs, medical assistants, and nurses for the theoretical sampling of participants. A theoretical sampling by re-defining appropriate sampling criteria during data collection and analysis for the identification of further participants was used in all three qualitative sub-projects from target 1.

Cooperating general practices and the PC unit of Hannover Medical School in Germany were informed about the study and made aware of the sampling criteria for patients and relatives. They supported the identification and invitation of potential participants by screening those of their patients and relatives who were currently undergoing treatment. In case the candidates matched the criteria, researchers from the study team contacted the individuals and applied for an interview. GPs, medical assistants and other professionals known from prior project cooperation as well as previously unknown potential participants were identified via theoretical sampling.

All interviews except for one done over the telephone were conducted face-to-face. An interview guide was specifically developed for this project (Table 1 (Tab. 1)). The interviews were simultaneously recorded on audio tape and transcribed verbatim, using a professional service. Additionally, a questionnaire assessing socio-demographic data was completed by all but one interview partner. The interviews took place in the respective workplace of the service provider, on the PC unit or in patients’ and relatives’ homes.

All participants were informed verbally and in writing about the content and purpose of the project. Every participant attended voluntarily, was unpaid, and signed an informed consent form.

### Data collection

The CIT [15], [16] was used to elicit participants’ descriptions and reflections about positive and negative examples of their experience with PC provision. The narrative examples were therefore intended to focus on:

1. the description of specific health care events (situation),
2. the observable behavior and actions of all concerned (behavior),
3. the context of the experienced events (context),
4. the consequences of the observed actions (outcome) [17].

To gain insights into these four aspects of an incident, predetermined questions were put forward in the interview guide. Interviewers used additional questions from a set of optional, predefined questions to encourage participants to elaborate on the experiences they have made when receiving or providing PC. The CIT was expected to uncover numerous critical incidents within one participant’s narrative.

The CIT is especially known to be helpful in assisting research participants to describe and reflect on specific incidents, and finding solutions for practical problems by an exact reflection of observed behavior [18], [19]. The rationale for choosing the CIT was the flexibility of the method and its ability to capture individual relevant health care factors from practice-based experiences that lead to excellent, undesirable or adverse PC provision.

### Data analysis

The analysis of synthesized data of the first phase of our study including three different qualitative sub-projects reached data saturation. Examples from the CIT analysis shown here do not aim at data saturation.

Quantitative demographic data and qualitative text material of the verbatim interview transcripts were understood as data basis for analysis. The demographic data was analyzed by means of descriptive statistics and frequency analysis with the statistical software IBM SPSS Statistics 24. The qualitative data analysis was supported by the software MAXQDA [20].

To answer the main research question, interview transcripts were screened for relevant text passages about the so-called ‘critical incident’ (CI). Within data analysis, a CI was defined and extracted from the interview text, if one of the following occurred:

1. reporting a lived experience in detail, including context information and related factors leading up to the experience and consequences from this CI;
2. reporting an experience from primary PC in an outpatient setting.

SSt extracted, defined, and counted CIs. After that, a thematic analysis of these extracted CIs was conducted independently by two researchers (SSt, HE). Both researchers allocated CIs to superior thematic groups (Table 2 (Tab. 2)) by asking: What is the actual incident here? In case of incongruence, the researchers discussed their allocation of CIs until they agreed on the thematic mapping of all extracted CIs. In the course of data analysis, CIs reporting similar contents were summarized in minor thematic categories such as ‘palliative care knowledge’ and finally abstracted to seven broader dimensions such as ‘Quality and outcome of care provision’ (Table 3 (Tab. 3)).

## Results

### Study population

15 out of 16 participants filled out a questionnaire on their socio-demographic data (Table 4 (Tab. 4)). Interview partners could participate without necessarily answering this questionnaire. One participant refused to fill out the questionnaire without giving reasons. Three of the GPs worked in separate joint practices, one GP worked solely in a single practice. Two GP practices treated between 1,000 and 1,500 patients per quarter, while the other practices treated more than 1,500 patients per quarter.

In two cases, we included a partner-relative relationship; one patient was single, living alone. One female relative was interviewed after the death of her husband. At the time of the interview, the other three relatives contained within the group were still in active contact with the PC patient.

### Critical incidents in palliative care provision

Within the 16 collected interviews of four patients (P), relatives (R), GPs, and medical professionals (MP) (two GPs’ assistants and two professional caregivers), a total of 130 positive and 150 negative CIs were uncovered and analyzed in detail (Table 3 (Tab. 3)). Only in the GPs, the number of positive CIs outweighed the negative ones. In all other three survey groups, more negative than positive CIs were reported (patients: 21/30; relatives: 31/44; GPs: 33/22; medical professionals: 45/54). The number of CIs reported per participant varied from 4 to 102.

In order to ensure anonymization of the study participants, the two GPs’ assistants and two professional caregivers will be named as MP in the following.

After careful thematic analysis, all described CIs were clustered into seven main content domains with several sub-domains (Table 3 (Tab. 3)).

### General formal conditions of care provision: financial regulations

One MP described a negative CI addressing the profound lack of financing PC in nursing homes, despite the clear expectation that nursing homes should deliver PC:

- **Situation:** *MPs are extremely committed to palliative care. In order for people to be cared for properly, a new framework has to be put in place in nursing homes as soon as possible to ensure adequate nursing care. That’s the main point.*
- **Context:** *A nursing home is simply the place where most people die. But in the meantime, the residents arrive so severely ill at the nursing home – not with oncological diseases, just comorbid people. And that is really challenging, caring for these people in the final stage. To make sure that a comfortable environment is created, that somebody is at the patient’s bedside, rather than the nursing staff rushing around the ward all the time.*
- **Behavior:** *Meanwhile the team has been educated well, so they say, “Okay, go to that room, the patient is not well at all, sit down there and stay for a while, [in the meantime] others will undertake your part of nursing [for the other residents]”.*
- **Outcome:** *Basic nursing, for example, happens on top, so to say. No pennies from the insurance company, the nursing home can’t account for anything, nothing at all. They do not get any money, none.*
  (MP_725a, 122–152)

### Quality and outcome of care provision: palliative care knowledge

For this MP, the negative CI about the lack of financing coincides with a lack of knowledge among colleagues. This is because the majority of nurses working in nursing homes are not specifically educated and trained in PC. The MP concludes that there is an urgent need for more knowledge transfer and training for a broader range of nursing homes’ employees:

- **Situation:** *There are 3 out of 8 colleagues that are skilled employees in palliative care. The others [5 colleagues] just have their normal training [in nursing care for the elderly]. With the addition of two nursing assistants, that’s the whole team.*
- **Context:** *There is a need for knowledge and that’s not only the case for the unpaid voluntary workers, but rather for everyone involved in a nursing home with integrated palliative care beds. A little education should be taking place for everybody, starting with the nursing staff and on to housekeeping, kitchen staff, and maybe even including the facility manager.*
- **Behavior:** *Hence, they simply know, “Okay, I can’t just gatecrash in the resident’s room and clean in there quickly, because the resident is in a poor condition”. Or how else come that the residents start asking me, “Could you hold my hand for a while?” – “How is it my turn to hold hands?”*
- **Outcome:** *Thus, it would be fine if that were to happen, but we haven’t got that far yet. *
  (MP_725a, 132-168)

### Communication: patient will and end-of-life wishes

Societal aspects of PC provision were discussed in the context of patients and relatives being informed or willing to be informed about the diagnosis and prognosis and (not) passing on this information among family members. In a negative CI, an MP felt PC delivery was hampered by a lack of openness to information:

- **Situation:** *Sometimes we don’t even know if the patient is informed or not [about the diagnosis], that’s the worst thing.*
- **Context:** *We received phone calls saying, “Yes, yes, but my dad is not supposed to know about it [meaning end-of-life stage]”, or nope, the wife doesn’t want her husband to know, then I say, “Then we actually can’t care for him”. When we get involved, we do insist that patients are made aware.*
- **Behavior:** *And we deal with that [palliative care] in rare cases because of the [patients’] huge distress. No one else can provide it [palliative care without patients being informed about diagnosis/prognosis], but we still do it... reluctantly.*
- **Outcome:** *And if we were to stay at the residents’ bedside, we would speak the truth. Anything else would make no sense. Everybody should question themselves,“[Do I want] to be fooled by my wife, husband, children [...]?”. The end is near, maybe someone wants to arrange something, perhaps you would like to put all your thoughts in order. I think you might be deprived of your life and the very last part of it, that’s a shame.*
  (MP_80c3, 161-163)

### Availability of care providers, structures, medication, and aids: access to inpatient hospice beds

In another negative CI, one MP described how missing local care resources for a patient and his wife led to a poor quality of dying and death and a difficult grieving process.

- **Situation:** *An elderly wife, [with a past medical history of] three herniated discs and two artificial knees, turning her obese 88-year-old husband back and forth in his bed, to the best of her knowledge, suffering from pain. And when he had passed away, she broke down.*
- **Context:** *She had absolutely no time for grieving, fell into a black hole. When the one person to care for is gone, how will life go on?*
- **Behavior:** *The support is simply missing then. Not necessarily by us, this [support] can also be given by other hospice or palliative care services. We live rural, it is quite difficult out here. There are good GPs and palliative care specialists, a few at least, but some patients fall through the cracks miserably.*
- **Outcome:** *Of course, an unacceptable end-of-life phase for the patient and the family is highly burdensome. They [the family] are shocked and under stress. This is very, very unsatisfying.*
  (MP_80c3, 359-361)

### Way of care provision: cooperative between health care providers

Participants of the interviews explicitly recommended PC when applied in a proactive, coordinated, continuous, individual, and GP-centered manner.

A patient described the negative experience of the lack of coordination of care:

- **Situation:** *I asked the specialist twice to send a fax to the oncology ward, but it failed twice. I called the oncology ward and asked if they had my blood test results, but on two occasions, they were not there.*
- **Context:** *Basically, they [oncology ward] wanted current blood tests to check if the infusion therapy could be initiated.*
- **Behavior:** *Now I go to my doctor once a week for blood tests. That works well with my doctor, so logically I go there to let them do it.*
- **Outcome:** *Because it works so seamlessly now, we have done this for a year now. *
  (P_a63c, 172-174)

### Way of care provision: proactive for patients

In contrast, a close relative of a terminally ill patient commended proactive PC delivery by the GP. Proactive PC was achieved by being informed about the anticipated disease trajectory and the preparation of the respective therapeutic measures which resulted in a positive CI:

- **Situation:** *And on Friday, the doctor was here for a talk and she looked after my dad again.*
- **Context:** *I realized then that it was going near the very end. I asked my questions, “What do I have to do when it comes to the very end?”*
- **Behavior:** *Then she put everything in place and wrote it down. And both, the two [GP and assistant] prepared the medication for every day, so I could decide what he [patient] needs. Then I administered a drug every six hours, you can’t do anything wrong like that.*
- **Outcome:** *Well, she [21] really took away my fear, and showed me who I can open up to, where you can get help or how they [relatives] can cope with it all finally.*
  (R_37dd, 22-26)

### Communication: patient will and end-of-life wishes

In a positive CI, a professional caregiver explained the positive consequences of individual, case-based PC delivery considering especially the patient’s and family’s wishes for end-of-life care and place of death:

- **Situation:** *There was a patient, [with a past medical history of] metastasized colon carcinoma. He developed an ileus, bile stasis, but besides that [he was] in a rather good physical condition. Because of the ileus [he was] at risk to die, a lot of complications, he couldn’t eat and drink, couldn’t stop vomiting, had paradoxical diarrhoea and insane pain.*
- **Context:** *His wife, in her seventies at the time, had already lost three of her children, but was basically energetic and didn’t want to give him away from home.*
- **Behavior:** *He went to the hospital once, the palliative care unit, for placing of a duodenal expiry probe.*
- **Outcome:** *He could eat and drink again. Of course, the disease wasn’t cured. He died probably two, three months after that procedure, but his wishes were considered until the very end, and most of those addressing his care were fulfilled.*
  (GP_6a60, 29-35)

### Availability of care providers, structures, medication, and aids

In a negative CI, the actual availability of a GP as an indicator for the presence of a superior care network was lacking reliability. This led to negative experiences for patients and relatives in a home-centered care situation. A wife described how she was denied help in an emergency, at night time, for her seriously ill husband:

- **Situation:** *The night was so bad for me, being completely alone.*
- **Context:** *I called the nursing care service. I said I need help, somebody to come along. And then they [one of the nursing care staff] said, “We can’t do anything to help now!”*
- **Behavior:** *And then I was pissed off, because they say they do 24/7 care, on call. And then I realized that I was on my own.*
- **Outcome:*** Then I called the emergency number [for a paramedic], they came, yes, and administered an injection to calm him down.*
  (R_7e67, 19)

Besides communication, the individual care network that is developed for patients was highly valued in a positive CI if congruent with individual patients’ needs. A patient described how he felt about the preventative setup of a (senior) medical alert system in his private home:

- **Situation:** *If you are at home and fall, you can get help by this [lifeline or medical alert system]. But you might have to wait quite a while, depending on what the emergency is.*
- **Context:** *I would describe it as a security system and then you are technically able to communicate with the people [from the medical alert system]. And if it [being safe at home] won’t work, they [paramedics via the medical alert system] come to your home and look after you.*
- **Behavior:** *I let it ring, the people would answer acoustically, I could tell them what is wrong or if the emergency call was a mistake. And the next step would be to send someone of their staff over.*
- **Outcome:** *But fortunately, nobody of the staff had to come over to our place.*
  (P_8af6, 8-34)

On the other hand, if the care infrastructure does not match the patients’ and relatives’ needs, a PC situation at home can be experienced as inadequate. A negative example of missing aids and appliances at home after a discharge from hospital, described by a patient’s relative, illustrates this issue:

- **Situation:** *Patients will be discharged from hospital. “Sent home for seven days.”*
- **Context:** *We have needs for a nursing bed, a hoist, various aids, but they would have to be at home beforehand, before coming home. The application for a care package was done by a social worker in the hospital. I was forced to pay in advance, not knowing if my health insurance was going to cover the costs at all.*
- **Behavior:** *I ordered a hoist via eBay then.*
- **Outcome:** *Yes, it [the hoist] is at home now. But now it’s too late anyway. As we finally got the financing accepted [from the health insurance], we were back here [in the hospital] again.*
  (R_86a5, 11-82)

### Quality and outcome of care provision: quality of patient care

A patient receiving PC by a GP illustrates, in a negative CI, how she experienced time pressure and disregard in a GP practice and how she and the GP dealt with their conflict:

- **Situation:** *Then we arrived at her practice and she [the GP] saw us. She used to say, “I have no time.” And then, “Yes, come and see me tomorrow at 8 am and I will make time tomorrow.”*
- **Context:** *On the next day, 8 am, I was there, I requested only a few minutes [of the doctor’s time]. And then she looks at the clock, “Oh, you know, I have got patients waiting for blood samples.” You ask once, you ask twice, you ask a third time... and you always hear, “No, I don’t have any time.”*
- **Behavior:** “*But today is Wednesday”, I say, “there are no blood samples today, that happens only on Mondays”. And I said to her, “You know what, if I am getting on your nerves, you can be open with me and tell me that I am stupid.” And then she [the doctor] got annoyed.*
- **Outcome:** *No, I did not end it [doctor-patient-relationship], I returned, but I distanced myself.*
  (P_e37f, 103-105)

A GP described the immediate symptomatic improvement of a patient who was holistically cared for by a second PC specialist and herself, which resulted in a very positive experience:

- **Situation:** *We took over a patient from a GP.*
- **Context:** *And he was a patient suffering from end-stage COPD, who additionally had a pulmonary embolism and in consequence a failure of a large part of his lung. The whole hallway, the entrance area, was full of oxygen bottles and the man was lying in a small room. The man had his oxygen running at 14 litres.*
- **Behavior:** *So we came there and slowly got to know each other. We explained everything during our talk, for a while, and we touched upon his matters.*
- **Outcome:** *The oxygen was lowered to 4 litres. This was very impressive, that’s when we noticed this had a psychosocial component.*
  (GP_0cc8, 7)

## Discussion

The majority of international work on PC provision by GPs considered the perspectives of GPs, but did not integrate the views from patients, their relatives, and other medical professionals, who are all part of PC delivery.

What do positive and negative incidents in primary PC show us, and what can we learn from patients’, relatives’, GPs’ and medical professionals’ experience in their primary PC? And, in consequence, what aspects of care provision lead to successful or undesirable PC?

The current literature states structural determinants of the health care system, such as lack of knowledge and PC skills, as hurdles for PC provision [2], which correlated strongly with our results from the experiences analyzed in the study at hand. MPs noticed that nursing homes nowadays have become a place of care and inevitably of death for many patients. Unfortunately, the lack of knowledge, education, and PC training represent great obstacles to high-quality PC provision. The care delivered in nursing homes is primarily assigned to generalist outpatient care in Germany, because mainly GPs attend to patients in nursing homes. For this generalist PC, no specific training is necessary, in contrast to specialist PC. The authors conclude that with the increasing professionalization of PC as a medical discipline [7], the standards of care and expectations regarding the quality of care for the dying and their families have risen [21], and PC providers are in urgent need of accessible targeted training to gain this up-to-date PC knowledge. This concern goes along with a need for adequate financing of PC performance for GPs and other care providers who were originally not trained to provide specialist PC, but to cover basic PC. The heavy workload and time pressures of GPs, criticized by a patient in a negative CI, seem to be contrasted with the time resources needed for comprehensive and holistic PC provision as described in a positive CI by a GP.

The particular manners of service provision, such as coordinated care which leads to positive PC experiences for many study participants, is also partly acknowledged in the literature. Mentioned key features of coordination of care were good communication and trust among colleagues in collaborative working models [12], [22], [23], [24]. In contrast, missing coordination and a resulting deficit of care for patients were assessed as very negative by interviewees [21]. In line with our findings, the literature stresses the significance of identification and inclusion of (over-)burdened relatives and their co-care for the success of generalist outpatient PC [25].

Particularly significant was the result that GP-centered care at the end of life was highly desired by all participating health care providers, patients, and relatives. This model has already been established successfully in England and the Netherlands, where patients see their GP as first point of contact for most health concerns. The GP works as a gatekeeper, before patients are referred to specialists. International experience also shows that GP-centered care at the end of life, especially when additional out-of-hour services are provided, leads to decreased numbers in hospital and emergency admissions [26], [27]. An evaluation of weekend face-to-face inpatient assessments by hospital specialist PC services showed that visits were mostly valued as highly appropriate and necessary. Essentially no misuse was detected [28]. Although a GP-centered care system has been tested to reduce health care costs in Germany, it could not be established for the long term [29]. Successful PC was experienced when the GP had a clear responsibility and was the first contact for patients and relatives. Separation of responsibility was identified as a significant barrier to successful continuity of care in the international literature [30], [31].

Study participants highlighted the need for a framework of care that is well adapted for patients and their relatives. These time-consuming demands are often experienced to be in conflict with the high GP caseload, and this results in competing priorities [12], [32], [33]. Requirements such as an out-of-hours availability for patients and relatives can be incompatible with other demands for a GP in everyday practice [34]. Other negative PC experiences, such as a lack of regional or local care infrastructure, are hard to address, although teamwork and cooperation with other PC service providers could unburden the capacity of GPs.

### Strength and weakness of the study

The study was, as predefined and accepted from the study application, limited to a rather small number of participants. Additionally, only 4 participants for each perspective were chosen, so that data saturation within this one sub-project was not reached. However, almost each participant reported numerous CIs. In addition, one CI often contained numerous detailed CIs, so that the actual number of analyzed CIs was much higher than the number of interviews. This was a valuable lesson. We identified key issues from multiple perspectives without aiming at data saturation within the assessed CIs. The strength of the study lies in highlighting the salience of certain factors contributing to PC delivery experienced as highly positive or negative from four different perspectives.

CIT as a method was perceived well by GPs and medical professionals. The use of CIT with patients and relatives was partly hampered due to i) a strict separation of positive and negative experiences, and ii) reporting events solely taking place in primary PC that could not always be achieved. In addition, a single assessed CI interview did not focus on the addressed key setting of the study, the outpatient care setting, but on experiences from an inpatient PC unit. It was not included in the analysis. The fact that only GPs reported more positive CIs than negative ones could be due to the fact that GPs evaluate their own work here.

During the interview analysis, certain contents could not be clearly placed into the four aspects of situation, behavior, context, and outcome. While applying our method, it became apparent that certain contents of a reported CI could be allocated into more than one of the aforementioned categories.

The authors are aware of these limitations. Nevertheless, the results allow valuable insights into factors determining the delivery of primary PC in everyday routines of GPs. Integrating this CIT sub-project into the broader concept of the multi-stage mixed-methods study ALLPRAX should compensate for these limitations.

## Conclusions

The results of this analysis raise awareness for aspects that lead to successful or undesirable PC experiences in practice, seen from different perspectives. They open up potential for improvements in primary care to support the care of those in their last phase of life. From the CIs, it appears to be central to target the improvement of professionals’ PC education and knowledge, feasibility of out-of-hours service, better coordination of care at the interface with other care providers, and measures to better deal with high workload and time pressure.

## Abbreviations

- BMBF: German Federal Ministry of Education and Research
- CI(s): critical incident(s)
- CIT: Critical Incident Technique
- GP: general practitioner
- MP: medical professional
- P: patient
- PC: palliative care
- R: relative

## Notes

### Authors’ contributions

SSt analyzed the data and wrote the draft manuscript. HE contributed substantially to conception and design of the draft and to the editing of the manuscript. OK was involved in the translation of results and language editing. NSch enhanced the quality of the manuscript by revising it critically for important intellectual content, based on his longstanding expertise in the social sciences, public health research, and primary palliative care.

All authors have given their approval to the final version of the manuscript.

### Ethical approval

The study was approved by the ethics committee of Hannover Medical School (No. 7260, February 14, 2017) before the project started.

### Informed consent

We confirm that informed consent has been obtained from all participants for the publication of individual patient data.

### Availability of data and materials

The datasets used and/or analyzed during the current study are available from the corresponding author on reasonable request.

### Trial registration

The parent project ALLPRAX is registered in the German Clinical Trials Register (Registration No. DRKS00011821; December 04, 2017) and the German Register of Health Care Research (Registration No. VfD_ALLPRAX_16_003817; March 30, 2017).

### Competing interests

The authors declare that they have no competing interests.

### Funding

This study is funded as a junior research group by the German Ministry of Education and Research within the program for enhancing infrastructures in health services research (Grant No. 01GY1610). The funding body does not have any influence on the design of the study, the collection, analysis, and interpretation of data, or in writing the manuscript.

### Translation

Interviews were conducted in German and translated into English by the authors.

### Acknowledgments

We sincerely thank all participating patients, relatives, medical assistants, nurses, and GPs for supporting this project. We would also like to thank Axel Poniwerski for his valuable assistance with English editing of the manuscript. The financing of ALLPRAX (BMBF FK 01GY1610) by the Federal Ministry of Education and Research is greatly acknowledged.

## Figures and Tables

**Table 1:**
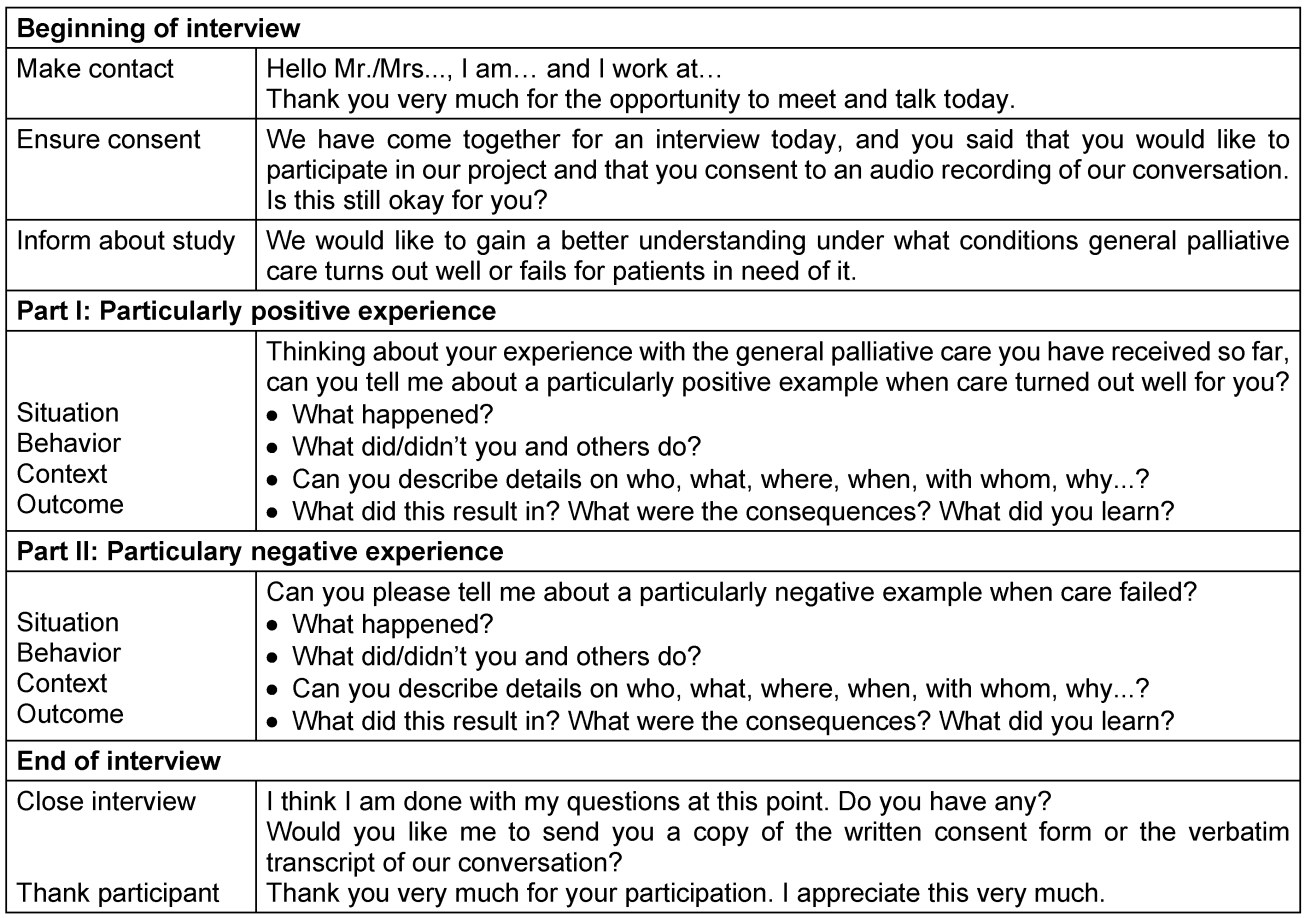
Exemplary interview guide for patients

**Table 2:**
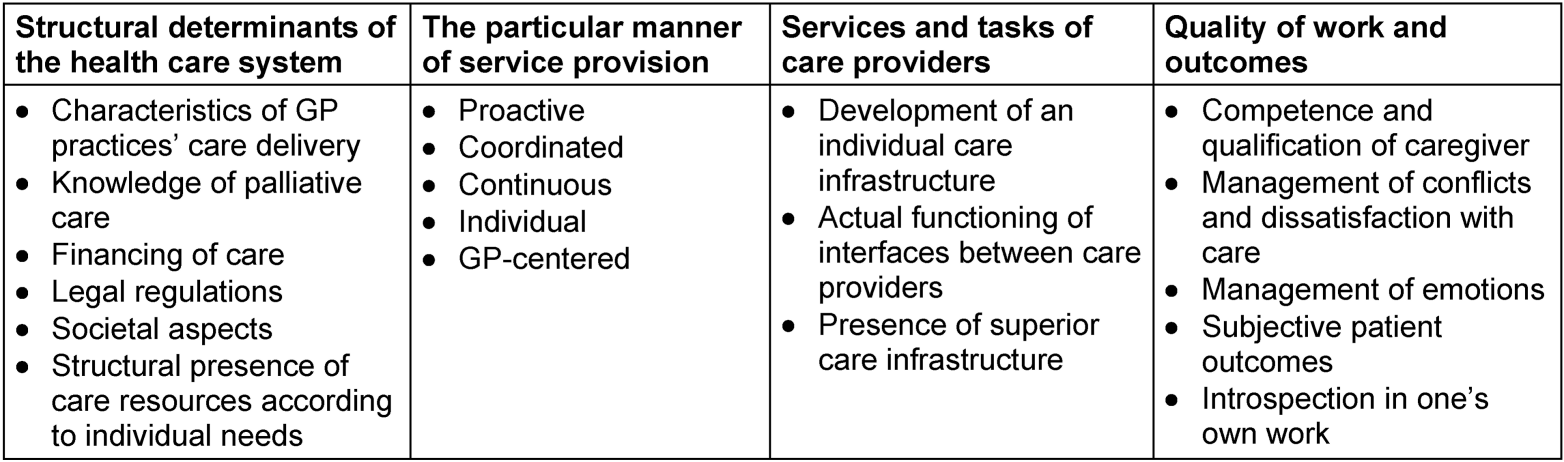
Structure of four superior content dimensions and inferior thematic categories of CIs

**Table 3:**
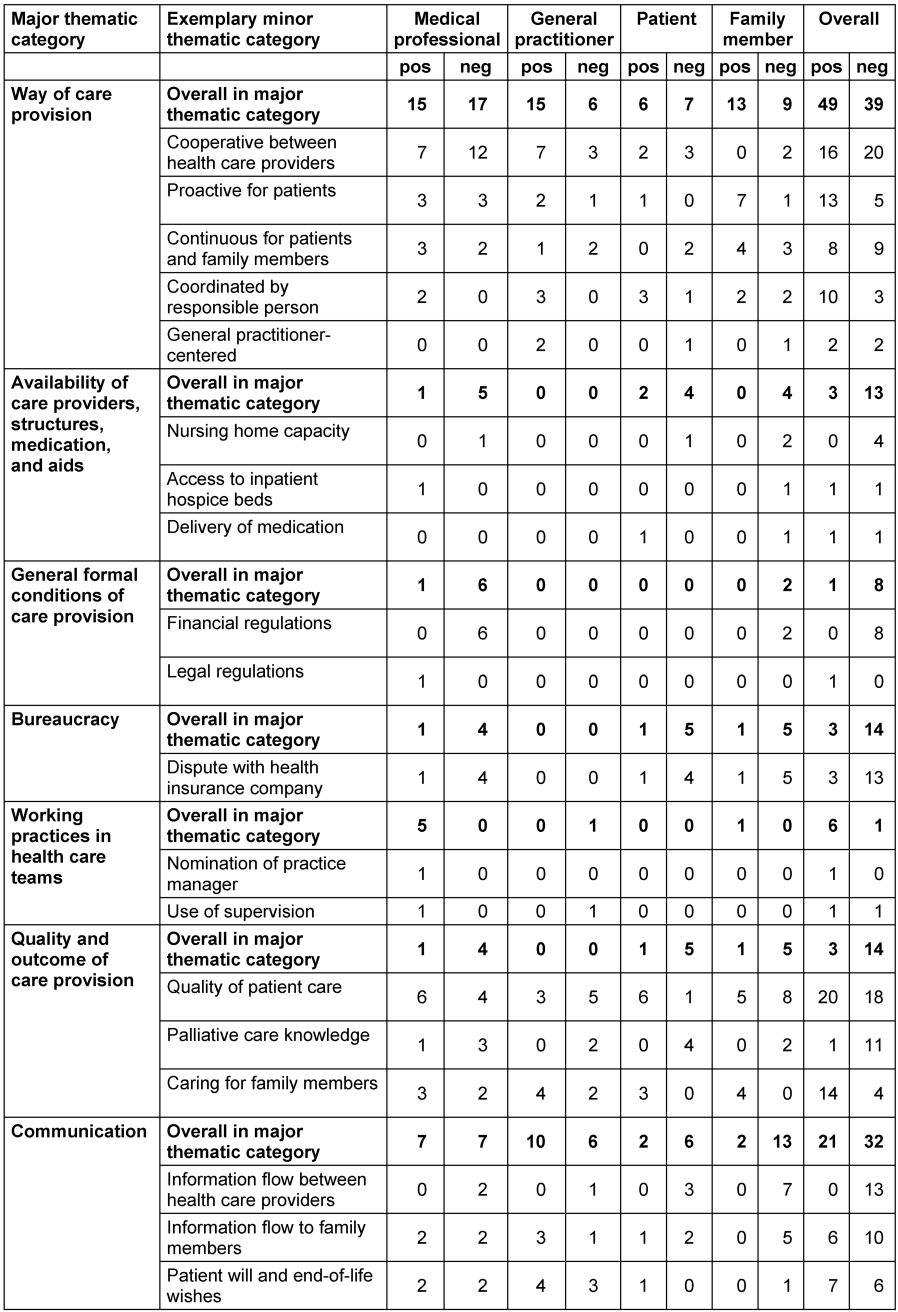
Absolute number of CIs in each participant group allocated to major and exemplary minor thematic categories

**Table 4:**
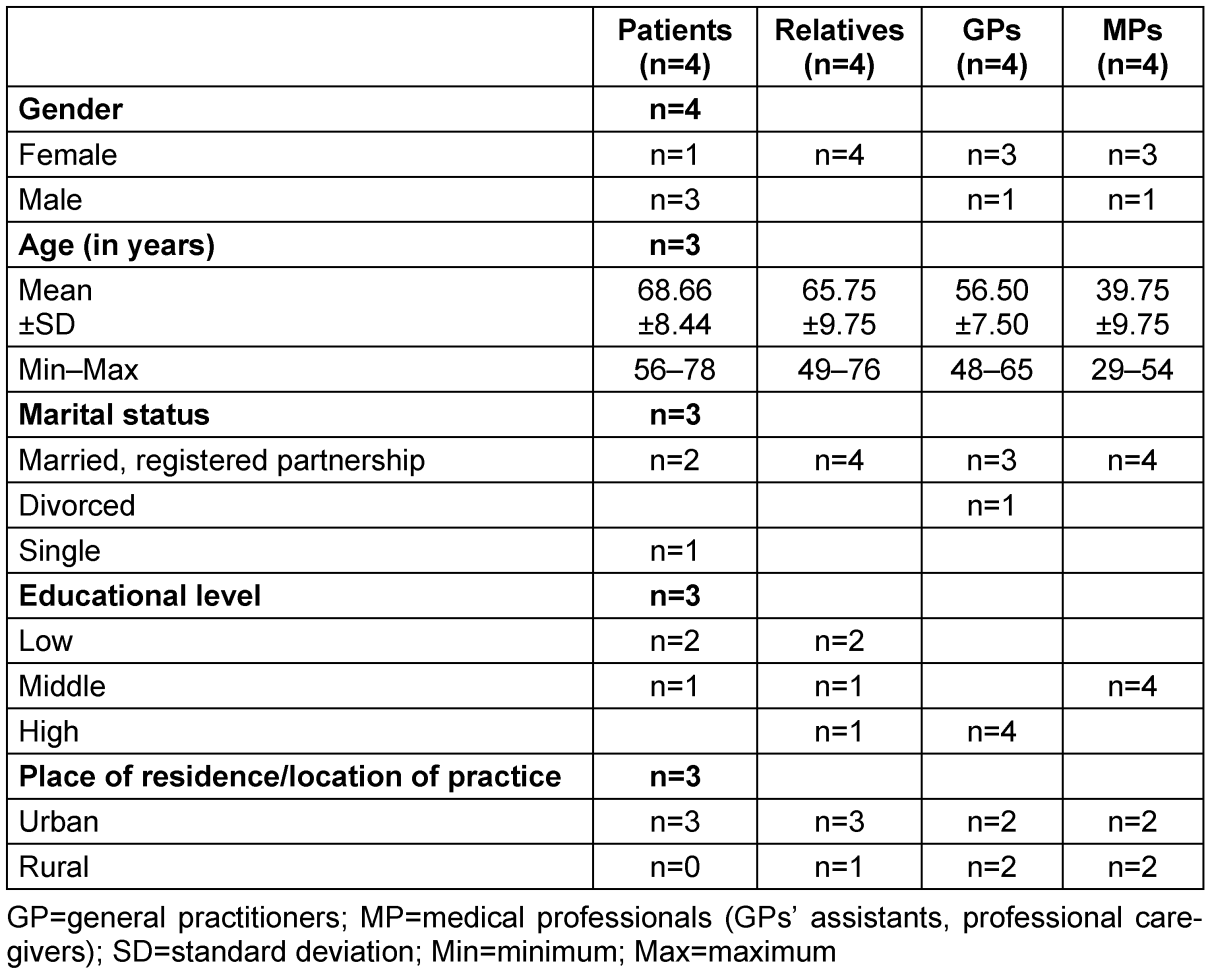
Sociodemographic data on study participants
